# Preclinical Evidence and Mechanism of Xingnaojing Injection for Cerebral Ischemia: A Systematic Review and Meta-Analysis of Animal Studies

**DOI:** 10.1155/2018/9624175

**Published:** 2018-11-15

**Authors:** Rong Ma, Xiao Ma, Jianxia Wen, Jian Wang, Qian Xie, Nian Chen, Taiwei Dong

**Affiliations:** College of Pharmacy, Chengdu University of Traditional Chinese Medicine, Chengdu 611137, China

## Abstract

**Objectives:**

Cerebral ischemia can cause severe harm to people's health with the characteristics of high incidence, high disability, and high mortality. Xingnaojing injection (XNJI) is widely used in the treatment of cerebral ischemia. The aim of this review is to evaluate the efficacy and mechanism of XNJI in animal models of cerebral ischemia.

**Methods:**

Total seven electronic databases in English or Chinese (CNKI, Wanfang, VMIS, PubMed, MEDLINE, Embase, and the Cochrane Library) about most experiments and studies which came out before June 2018 of XNJI for cerebral ischemia have been searched. Data extraction, quality assessment, and meta-analysis are conducted according to the Cochrane standards and RevMan 5.3 software.

**Results:**

We have identified 23 eligible studies and made a meta-analysis based on these studies. Meta-analysis shows that XNJI contributes significantly to reduction in neurological deficit score (*P *= 0.0002, MD = −1.25, 95%* CI*: −1.92, −0.58) compared with the control group of cerebral ischemia. Subgroup analytic results demonstrate that XNJI has been more effective in animal model of cerebral ischemia-reperfusion injury (*P *= 0.009, MD = −1.35, 95%*CI*: −2.36, −0.34) than that of permanent cerebral ischemia (*P *= 0.0002, MD = −1.08, 95%*CI*: −1.66, −0.51). Compared with control group, XNJI could remarkably reduce cerebral infarction area (*P *< 0.00001, MD = −14.98, 95%*CI*: −21.36, −8.59), brain edema (*P *< 0.00001, MD = −4.64, 95%*CI*: −5.38, −3.90), and neuronal cell apoptosis (*P* < 0.0001, MD = −12.21, 95%*CI*: 18.05, −6.37). Meanwhile, the meta-analysis shows that XNJI has a significant anti-inflammatory effect, and the levels of TNF-*α*, IL-6, and IL-1*β* are significantly reduced by XNJI (*P* = 0.001, MD = −4.13, 95%*CI*:−6.68, −1.58;* P* < 0.00001, MD = −119.23, 95%*CI*: −138.04, −100.43;* P* = 0.21, MD = −228.69, 95%* CI*: −586.20, 128.83). Additionally, XNJI could raise the body's antioxidant function and the level of SOD and GSH-Px (*P* = 0.002, MD = 53.02, 95%* CI*: −20.52, 85.78;* P *= 0.01, MD = 8.65, 95%* CI*: 1.77, 15.48) and decrease the level of MDA (*P* < 0.00001, MD = −4.16, 95%* CI*: −5.50, −2.82).

**Conclusion:**

XNJI might be effective in cerebral ischemia by regulating oxidative stress and inflammatory reaction.

## 1. Introduction

Cerebral ischemia is commonly known as ischemia stroke, accounting for more than 80% of stroke cases. It seriously harms people's health with high incidence, high disability, and high mortality [1, 2]. As reported, ischemia stroke is a major cause of global mortality and morbidity [3–5]. With the fast pace of modern life and the gradual increase of modern stress, the rate of stroke has increased year by year, and it is tending to encroach on younger adults. Moreover, stroke leads to varying degrees of functional impairment for survivors, which brings serious burden to family and society. It is believed that the increasing global impact of ischemia stroke in the few decades will certainly affect the healthcare in several developing countries, including China.

Cerebral ischemia is caused by cerebral vascular occlusion induced by many reasons. Cerebral vascular occlusion leads to cerebral ischemia, hypoxia, and a series of pathological damage to brain. The physiological of cerebral ischemia is generally thought as a rapid cascade reaction with multilink and multichannel characteristics [6, 7]. In this event, the local steady state is broken and the pathological change is produced with the mix of excitotoxicity, oxidative stress, intracellular calcium overload, inflammatory reaction, cellular swelling, and eventually apoptosis or necrosis. In particular, the injury of blood brain barrier (BBB) structure and function is the key process. When in reperfusion, inflammatory factors could permeate through the damaged BBB into the brain. Moreover, many of the original harmless substances, such as oxygen molecules, will also be harmful via BBB. These factors lead to brain injury, and the brain injury became more serious during reperfusion. This situation causes great difficulty in the treatment of cerebral ischemia patients [8, 9].

Currently, the only effective drug for stroke which has been approved by Food and Drug Administration (FDA) is tissue-type plasminogen activator (t-PA), which is thrombolytic therapy by intravenous injection. It can achieve recanalization of blood flowing that is considered to be the most direct and effective treatment method. However, the drug must be used after a short period of limited stroke medication and secondary risk of bleeding; to a certain extent, it limits the clinical application of thrombolytic therapy [10]. Therefore, the new strategy treatment for stroke as well as long-term brain protection of new drugs is still the field of cerebral ischemia injury prevention and research focus and objectives [11]. And western medicine is increasingly recognized as failing to achieve the desired goals in the treatment of complex diseases, whereas traditional Chinese patent medicine (TCPM) can make substantial improvements in these diseases based on traditional Chinese medicine wholism.

Xingnaojing injection (XNJI), an effective TCPM, is derived from a classic Chinese emergency prescription named An Gong Niu Huang pills. An Gong Niu Huang pills is from “Treatise on Differentiation and Treatment of Epidemic Febrile Diseases” written by Wu Tang in the Qing Dynasty, which is widely used to cure various acute cerebrovascular diseases with good effectiveness. XNJI is produced by the secondary distillation of steam from the following four herbs: artificial* Moschus*, synthetic* Dryobalanops aromatica Gaertn. F., Gardenia jasminoides Ellis,* and* Radix Curcumae. Moschus*.* Radix Curcumae. Moschus* is the preferred medicine to rescue with aromatics, and combination with* Dryobalanops aromatica Gaertn. F.* could enhance the effect of resuscitation [2, 12]. In recent research, it demonstrated the characteristics and advantages of multitarget, multicomponent and multichannel regulation [12]; XNJI could directly act on the BBB permeability in nerve center by inhibiting inflammatory factors effectively [13–17]. Moreover, it could regulate the effect on cognitive function and antioxidant free radicals [18, 19], alleviate encephaledema, and ameliorate the ischemia, anoxic state anticell autophagy, and apoptosis in brain [16, 20–24].

**Figure 1:**
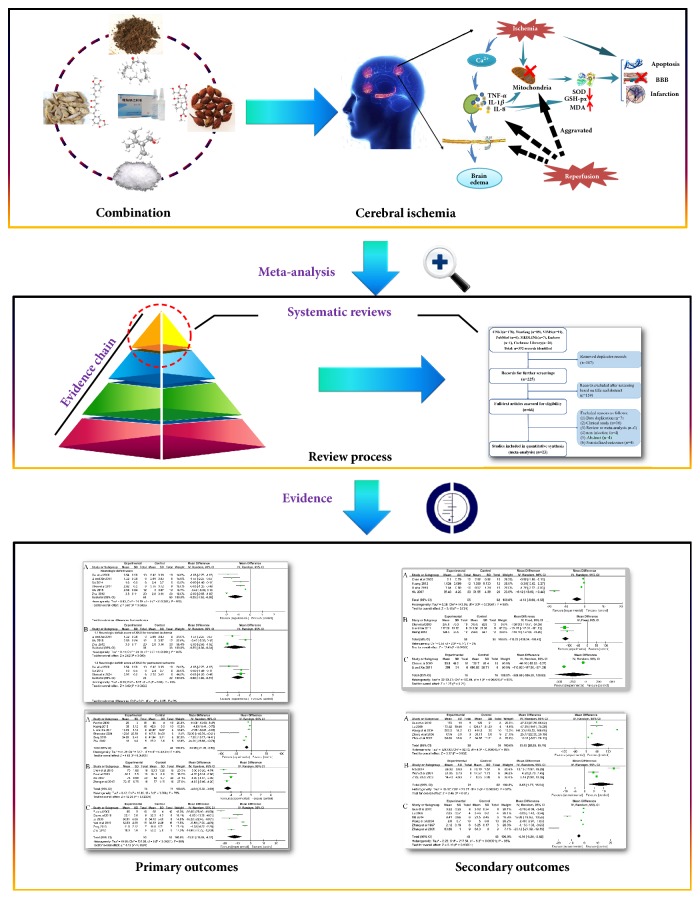
The meta-analysis process of the literature.

At present, clinical meta-analysis demonstrates that XNJI could promote the recovery of neurological function in patients with cerebral ischemia and reduce the cerebral infarction area [25]. And, there are many studies reporting the mechanism of XNJI on cerebral ischemia; however, there is no publication to summarize the mechanism of Xingnaojing treatment of cerebral ischemia. Therefore, we performed a systematic review and meta-analysis of experimental animal studies to gain a better understanding of the effect and mechanism of XNJI on cerebral ischemia and to explore its mechanism further (Figure 1).

## 2. Methods

### 2.1. Literature Search

Literature filtrating was conducted independently by two investigators (Rong Ma and Jianxia Wen), including Chinese National Knowledge Infrastructure (CNKI), Wanfang Database, VIP medicine information system (VMIS), PubMed, MEDLINE, Embase, and the Cochrane Library from the inception to June 2018. The following terms searched were used individually or in combination: “Xingnaojing injection” OR “XNJI” AND “stroke” OR “cerebral ischemia” OR “cerebral ischemia-reperfusion”.

### 2.2. Inclusion and Exclusion Criteria

The inclusion criteria are as follows: (i) the experiment is based on animal model of cerebral ischemia; (ii) treatment group receives the XNJI only; (iii) the included studies contain control group and XNJI treatment group; (iv) studies must include one of the defined outcome measures. The primary outcome measures are as follows: neurological deficit score, brain edema, cerebral infarction area, and neuronal apoptosis; the second outcome measures: tumor necrosis factor-*α* (TNF-*α*), interleukin-6 (IL-6), interleukin-1*β* (IL-1*β*), superoxide dismutase (SOD), glutathione peroxidase (GSH-Px), and malondialdehyde (MDA).

The studies were excluded, if they presented the following criteria: (i) clinical studies; (ii) treatment group without XNJI or combined with other agents; (iii) review and/or meta-analysis; (iv) the primary outcome measures and the second outcome measures which are not included in the literature; (v) duplicated publications; (vi) the article which has only an abstract.

### 2.3. Data Collection

The data extraction and quality assessment of the included studies were conducted independently by two investigators (Rong Ma and Jianxia Wen), and any disagreements were solved through discussion with corresponding author. We have extracted the following data from the included studies: (1) the first author's name and year of publication; (2) species of animals, animal sex, animal weight, numbers of animals in XNJI treatment group, and control group; (3) the model of cerebral ischemia (transient or permanent), the time of cerebral ischemia and reperfusion, XNJI dosage, and intervention duration; (4) primary and second outcome measures. And if the experimental group of animals in the study received various doses of XNJI, only the data of highest dose of XNJI was included.

### 2.4. Assessment of Methodological Quality

The methodological quality of the included studies was assessed based on the Collaborative Approach to Meta-Analysis and Review of Animal Data from Experimental Studies (CAMARADES) with 10-item quality checklist [26]: (1) publication in a peer-reviewed journal; (2) statement of temperature control; (3) random allocation to groups; (4) allocation concealment; (5) blinded assessment of outcome; (6) use of anesthetic without significant internal protection of blood vessel; (7) appropriate animal model (aged, healthy, diabetic, or hypertensive); (8) sample size calculation; (9) compliance with animal welfare regulations; (10) statement of potential conflict of interests. Each study was assessed and scored on a scale from 0 (lowest) to 10 (highest) points.

### 2.5. Statistical Analysis

All values of neurological deficit score, cerebral infarction area, brain edema, neuronal cell apoptosis, and the level of TNF-*α*, IL-6, IL-1*β*, SOD, GSH-Px, and MDA are considered as continuous data and analysed by RevMan 5.3 (2008 The Nordic Cochrane Centre). Outcomes were presented as mean difference (MD) with 95% confidence interval (*CI*) and the* I*-square (*I*^*2*^) statistic used to assess heterogeneity. If the* I*^*2*^ = 0, which suggested that there is no heterogeneity, fixed effect model would be used to perform a meta-analysis. If the* I*^*2*^ ≤ 50 % or* P* ≥ 0.1, it is considered minor heterogeneity with fixed effect model. If the* I*^*2*^ > 50% or* P *< 0.1, there is significant heterogeneity with random effect model.

## 3. Results

### 3.1. Study Identification and Selection

**Figure 2:**
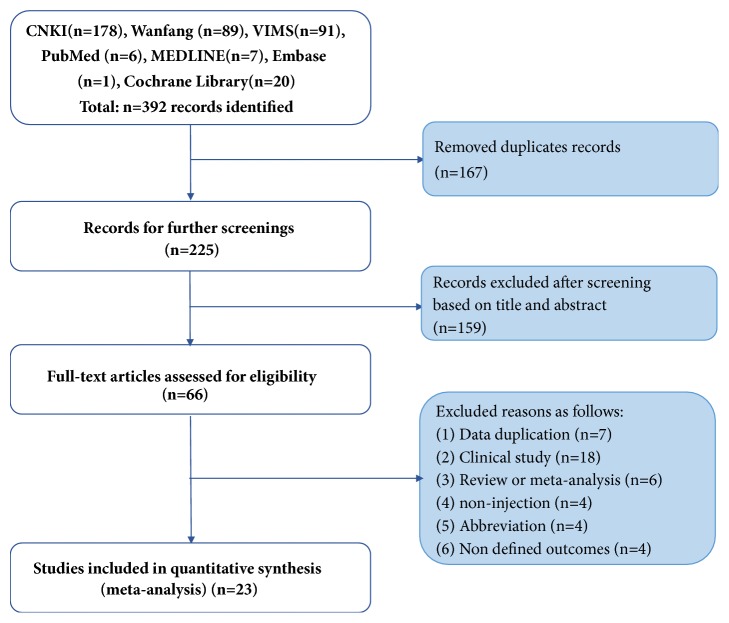
Flow diagram of the systematic review.

A flowchart of the study selection process is shown in Figure 2. The search has identified 392 relevant studies. However, we searched few specific studies from the literature search of the foreign language databases because XNJI is only used domestically in China currently. Ultimately, 23 studies with 578 animals were screened by title, abstract and full-text for further quality assessment [13, 16, 17, 20–24, 27–41].

### 3.2. Study Characteristics

A total of 578 animals (290 in the trial group; 288 in the control group) were included in the 23 studies. Sprague-Dawley (SD) rats or Wistar rats were used in most of the animal experiment; other animals such as mongolian gerbil, mice, and rabbits were used in a few experiment. Most of the research projects were male rats, both male and female, were also included. In the selected studies, the experimental animals were predominantly using intravenous or intraperitoneal injection in the dosage of XNJI 0.5 ~ 50 mL/kg and the same volume of normal saline as a control, and most of the dosing methods were administered in a single dose. Additionally, outcomes included neurological deficit score, brain edema, cerebral infarction area, neuronal apoptosis, serum levels of inflammatory factors (TNF-*α*, IL-6, and IL-1*β*), and the antioxidant level of body (SOD, MDA, and GSH-Px). There were also three articles which explored the impact of XNJI on BBB damage [20, 29, 34]. The detailed characteristics of the 23 articles are shown in Table 1.

### 3.3. Methodological Quality

All studies were published in a peer-reviewed journal. Seven studies described the temperature control [13, 29, 30, 32, 33, 37, 39]; eighteen studies described random allocation [13, 16, 20–24, 27–30, 32–36, 38, 40], and four studies reported the random sequence was gene rated by a random number table [29–31, 33]. None of the studies used either allocation concealment or blinded assessment of outcome. As for the use of anesthetics, chloral hydrate was used as anesthetic in nine studies [13, 20–22, 29–31, 33, 38], pentobarbital sodium was used as anesthetic in three studies [16, 32, 40], ethyl ether was used as anesthetic in two studies [23, 39], and isomo pentobarbital sodium, ethyl carbamate, and thiophene sodium were used as anesthetic in one studies, respectively [28, 36, 41]. Six articles did not report the detail of anesthetics [17, 24, 27, 34, 35, 37]. One study chooses rats with hypertensive [32], and others choose the healthy mouse, rats, or rabbits. Fifteen studies described a sample size calculation [16, 17, 20, 21, 24, 27–31, 33, 34, 36, 40, 41]. And, no studies reported compliance with animal welfare regulations or a statement of potential conflict of interests. Details of each experiment are shown in Table 2.

## 4. Effect of Interventions on Cerebral Ischemia

### 4.1. Primary Outcome Measures

#### 4.1.1. Neurological Deficit Score

**Figure 3:**
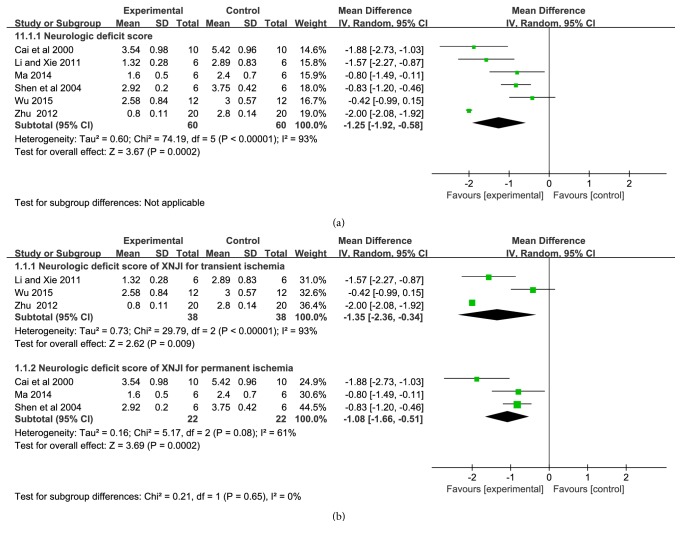
Forest plot of comparison: (a) neurological deficit score; (b) subgroup of XNJI on transient and permanent ischemia;* I*^*2*^ and* P* are the criterion for the heterogeneity test, ◆ pooled mean difference, —■— mean difference, and 95%* CI*.

Neurological deficit score was measured in seven studies with 120 animals. Significant heterogeneity occurred in index of neurological deficit score (*P *< 0.00001,* I*^*2*^ = 93%). Therefore, random effect model was adopted for meta-analysis. The MD with 95%* CI* of neurological deficit score was −1.25 (−1.92, −0.58), indicating that XNJI could significantly reduce the neurological deficit score compared with control group (*P*=0.0002) (Figure 3(a)). Subgroup analysis showed that XNJI got a remarkable effect on the models of ischemia-reperfusion [n=76, −1.35 (−2.36, −0.34),* P*=0.009] compared to permanent ischemia [n=44, −1.08 (−1.66, −0.51),* P*=0.0002] (Figure 3(b)).

#### 4.1.2. Cerebral Infarction Area, Brain Edema, and Neuronal Cell Apoptosis

**Figure 4:**
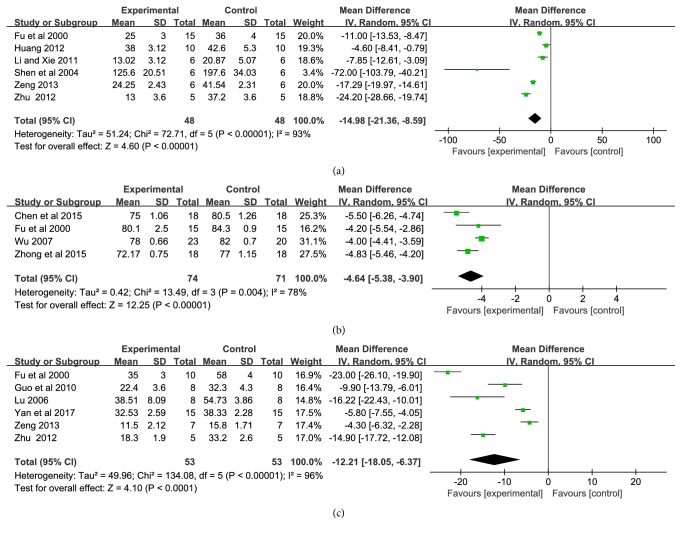
Forest plot of comparison: (a) cerebral infarction area; (b) brain edema; (c) neuronal cell apoptosis.* I*^*2*^ and* P* are the criterion for the heterogeneity test, ◆ pooled mean difference, —■— mean difference, and 95%* CI*.

Cerebral infarction area, brain edema, and neuronal cell apoptosis were measured in six studies [13, 21, 24, 33, 34, 37], four studies [16, 20, 24, 29], and six studies [21, 22, 24, 27, 33, 35] with 96, 145, and 106 animals, respectively. Significant heterogeneity appeared in cerebral infarction area, brain edema, and neuronal cell apoptosis (*P* < 0.00001,* I*^*2*^ = 93%;* P* = 0.004,* I*^*2*^ = 78%,* P *< 0.00001,* I*^*2*^ = 96%). Therefore, random effect model was adopted for meta-analysis. The MD with 95%* CI* of cerebral infarction area, brain edema, and neuronal cell apoptosis were −14.98 (−21.36, −8.59) (Figure 4(a)), −4.64 (−5.38, −3.90) (Figure 4(b)), −12.21 (−18.05, −6.37) (Figure 4(c)). The pooled analysis indicated that the cerebral infarction area, brain edema, and the neuronal cell apoptosis could significantly alleviated by using XNJI compared with control group(*P *< 0.00001,* P *< 0.00001, and* P *< 0.0001).

### 4.2. Second Outcome Measures

#### 4.2.1. Inflammatory Factor

**Figure 5:**
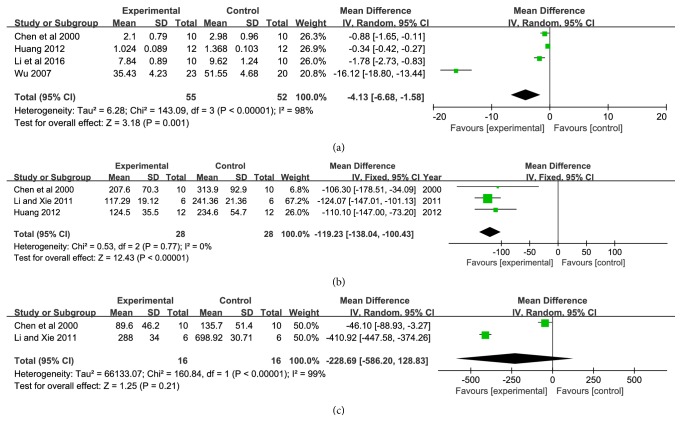
Forest plot of comparison: (a) TNF-*α*; (b) IL-6; (c) IL-1*β*.* I*^*2*^ and* P* are the criterion for the heterogeneity test, ◆ pooled mean difference, —■— mean difference, and 95%* CI*.

The effect of XNJI on TNF-*α*, IL-6, and IL-1*β* is summarized in Figure 5. Serum TNF-*α*, IL-6, and IL-1*β* level were measured in four [13, 16, 29, 34], three [13, 17, 34], and two articles [17, 34] with 107, 56, and 32 animals, respectively. Significant heterogeneity represented in index of TNF-*α* and IL-1*β* (*P* < 0.00001,* I*^*2*^ = 98%,* P* < 0.00001,* I*^*2*^= 99%). Therefore, random effect model was adopted for meta-analysis. The MD with 95%* CI* and* P* of TNF-*α* and IL-1*β* was −4.13 (−6.68, −1.58),* P *= 0.001; −228.69 (−586.20, −128.83),* P*= 0.21 (Figures 5(a) and 5(c)). In addition, there were two experiments which assessed the changes in IL-1*β*. There is no heterogeneity displayed in the meta-analyses of IL-6 (*P =* 0.77,* I*^*2*^ = 0%). Based on the statistical meta-analysis of fixed effect model, the MD with 95%* CI* and* P* of IL-6 was −119.23 (−138.04, −100.43),* P < *0.00001(Figure 5(b)). It demonstrated that XNJI could alleviate cerebral ischemia injury by inhabiting inflammatory factor in vivo significantly.

#### 4.2.2. Antioxidant Level

**Figure 6:**
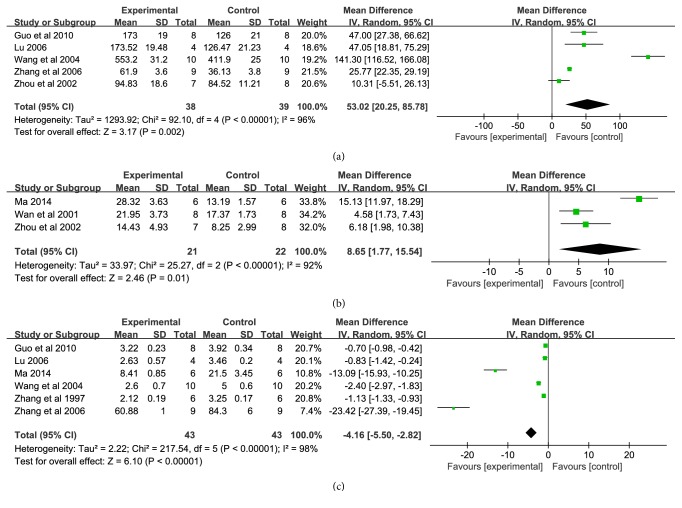
Forest plot of comparison: (a) SOD; (b) GSH-Px; (c) MDA.* I*^*2*^ and* P* are the criterion for the heterogeneity test, ◆ pooled mean difference, —■— mean difference, and 95%* CI*.

SOD, GSH-Px, and MDA level, the direct index of oxidation system of the body, were measured in five [22, 23, 35, 36, 38], three [32, 38, 39], and six articles [22, 23, 32, 35, 36, 41] with 77, 43, and 86 animals, respectively. Significant statistical heterogeneity was observed in all the three meta-analysis (*P* < 0.00001,* I*^*2*^ = 96%;* P* < 0.00001,* I*^*2*^= 92%;* P* < 0.00001,* I*^*2*^ = 98%). The MD with 95%* CI* of SOD (Figure 6(a)), GSH-Px (Figure 6(b)), and MDA (Figure 6(c)) was 53.02 (20.25, 85.78), 8.65 (1.77, 15.54), and −4.16 (−5.50, −2.82), respectively. It indicated that the XNJI could significantly increase the level of SOD, GSH-Px and decrease the level of MDA (*P* = 0.002,* P* = 0.01,* P* < 0.00001).

## 5. Discussion

### 5.1. Preclinical Evidence and Mechanism of Xingnaojing Injection for Cerebral Ischemia

The rapid development of modern technology makes many oral formulations of Chinese medicine developing as injections to meet the needs of modern medicine. XNJI is refined based on the traditional Chinese prescription named An Gong Niu Huang pills [42], which is widely applied in clinic for a variety of coma and cerebrovascular accident patients [43, 44]. It could alleviate quickly and effectively the clinical symptoms of cerebral ischemia such as hemiplegia, partial body sensory disorder, aphasia vomiting, and ataxia [45].

The pathogenesis of cerebral ischemia and reperfusion is a rapid cascade reaction. The accumulation of inflammatory cytokines in ischemia tissue is an important factor to aggravate cerebral ischemia and hypoxia. When cerebral ischemia-reperfusion occurs, cerebrovascular endothelial cells are activated. Meanwhile, platelets and immune cells release a large number of proinflammatory cytokines, including TNF-*ɑ*, IL-6, IL-1*β*, PAF (platelet activating factor), and complement activation products substance. TNF-*ɑ* is a vital role in the inflammatory network of cytokines and is considered as the trigger medium of a systemic inflammatory reaction [46]. It can directly inhibit vascular endothelial cell function, increase vascular permeability, decrease circulatory resistance, and induce cytokines and adhesion molecules release like the waterfall, resulting in inflammatory damage cascade amplification effect, finally, resulting in wall thickening, stenosis, and cerebral infraction [21, 22]. Through the meta-analysis, we found XNJI had significant anti-inflammatory effect. Compared with control group, the TNF-*α*, IL-6, and IL-1*β* levels of XNJI group were lower by 4.13, 119.23, and 228.69 ng/L (Figure 5). In addition, the results showed that the* I*^*2*^ value of IL-6 was 0%, suggesting a relatively reliable therapy.

Oxidative stress is another reaction after cerebral ischemia, and the function of free radical scavenging system in the body decreased during cerebral ischemia. The endogenous antioxidant systems are unbalanced and produced large amounts of free radicals, leading to the peroxidation of lipid, protein and nucleic acid, and the biochemical alteration (SOD ↓, GSH-Px ↓, and MDA↑), and further led to BBB disruption with secondary vasogenic edema, activation of apoptosis, and brain infarction [47, 48]. Therefore, the production and release of oxygen free radicals are the key steps that cause cerebral injury. Scavenging oxygen free radicals is an important way to treat cerebral ischemia-reperfusion injury. In theory, block oxidative stress or act on the key molecules could reduce cerebral ischemia-reperfusion induced oxidative damage. Our data provide evidence that XNJI could improve the body's antioxidant function after cerebral ischemia-reperfusion (Figure 6), suggesting that XNJI may reduce the damage of cerebral ischemia by regulating inflammatory and oxidative stress.

The important outcomes of cerebral ischemia and reperfusion injury are neurological deficit score, cerebral infarction area, brain edema, and neuronal cell apoptosis. Inflammatory, oxidative stress, and destroyed BBB could be cause and effect by each other and lead to cerebral edema, cell necrosis and activation of apoptosis [49], and brain infarction, which were manifested as neurological dysfunction in clinic [9]. In the animal experiment, it was tested by neurological deficit score. STAIR (2009) states that [50] multiple indicators should be selected in the study of cerebral ischemia drugs, which should include infarction area and neurological function; this emphasized the importance of behavioral outcomes due to its close relationship to late survival rate clinically. Functional recovery of clinical stroke patients is a major endpoint indicator [51, 52]. Evidence showed the beneficial of XNJI on neurological function (Figure 3) and cerebral infarction area (Figure 4(a)), which is consistent with clinical reports [25]. At the same time, subgroup analysis showed that XNJI had a better effect on the model of cerebral ischemia-reperfusion injury than the model of permanent cerebral ischemia. However, the heterogeneity still existed that may be related to different dose and duration of XNJI. Additionally, there were a study which had reported that XNJI have a significant effect on ultrastructural of brain when ischemia-reperfusion [17] and three studies reported the alleviation of XNJI on BBB damage [20, 29, 34]; however, we did not conduct a meta-analysis due to its quantitative limitations. The destruction of BBB plays an extremely important role in the process of cerebral ischemia. It is the destruction of BBB that causes the originally innocuous substance into the brain tissue to become harmful substances. Therefore, it is recommended that the BBB research should make a consideration in the further. Moreover, most of the studies included in this study are preventive one-time administration; however, cerebral ischemic stroke is mostly treated with XNJI after onset for one week, clinically. Therefore, it is recommended that study with therapeutic administration and longer administration may be considered in the further.

### 5.2. Strength

Preclinical efficacy experiments are typically cited to justify the initiation of clinical trials. Our findings contribute to the literature on preclinical design and reinforce our exploratory analysis for the mechanism of XNJI against cerebral ischemia. Moreover, they could eliminate unnecessary repetitive tests and contribute to further study in animal experiments to increase the likelihood of success in future clinical trials. Furthermore, as a traditional Chinese medicine, XNJI is a relatively safe drug. Therefore, it may play a potentially greater role in clinical practice in the future.

### 5.3. Limitations

The average quality score of studies was 4.5. Many studies have failed to describe their methods in detail, such as randomized trials, blind evaluation of results, and assignment of hidden. Therefore, we recommend that all research published in China should follow a guideline similar to the Consolidated Standards of Reporting Trials (CONSORT) Statement for clinical studies [53]. These strategies will promote the identification and use of many TCPM (e.g., XNJI) outside China.

In addition, there are some deviations caused by the following reasons: first, different animal species, drug dose, duration of administration, and method of administration resulted in some deviation. Second, animal models used in most studies are healthy; however, patients with cerebral ischemia are often associated with diabetes, high blood pressure, hyperlipidemia, and the like. Third, our search strategy includes only Chinese and English databases, leading to certain deviations. Thus, the results should be interpreted with caution.

## 6. Conclusions

Based on the results of this meta-analysis, the effect of XNJI on cerebral ischemia is encouraging. XNJI may be a promising method to alleviate ischemia-induced brain damage by regulating oxidative stress and inflammatory reaction. Considering being accepted far and wide by practitioners, the experiments with more rigorous experimental design and stronger quality control are required.

## Figures and Tables

**Table 1:** The characteristics of the included studies.

**Study(year)**	**Special/strain**	**Sex**	**Weight (g)**	**N(T/C)**	**Model**	**Index **	**Intervention**		**Outcome measures**
						**isch/rep(h)**			
							**Control**	**Treatment (duration)**	
Yan et al. 2017	Rat/W	M	250± 20	24(12/12)	P	2/24	i.p. NS	i.p. XNJ 5 mL·kg^−1^·d^−1^(1d)	NDS, NCA

Li et al. 2016	Rat/W	M	NR	20(10/10)	P	1.5/72	i.p. NS	i.p. XNJ 20 mL·kg^−1^·d^−1^(7d)	NDS,TNF-*α*, CIA

Chen et al. 2015	Rat/W	M	200~250	36(18/18)	P	0.16/48	i.p. NS	i.p. XNJ 10 mL·kg^−1^·d^−1^(1d)	BE, BBB

Wu 2015	Rat/W	M	250 ±50	24(12/12)	P	2/70	i.p. NS	i.p. XNJ 50 mL·kg^−1^·d^−1^ (5d)	NDS

Zhong et al. 2015	Rat/W	M	200~250	108(54/54)	P	0.16/72	NR	i.p. XNJ 10 mL·kg^−1^·d^−1^(3d)	BE, BBB

Huang et al. 2014	Rat/W	M	280~300	20(10/10)	P	0.3/0.3	i.p. NS	i.p. XNJ 2 mL·kg^−1^·d^−1^(1d)	CIA

Ma 2014	Rat/SD	M	200 ±20	12(6/6)	T	24/-	i.p. NS	i.p. XNJ 3 mL·kg^−1^·d^−1^, 3times (1d)	NDS, GSH-Px

Zeng 2013	Rat/SD	M	280~300	20(10/10)	P	2/24	c.i.v NS	c.i.v. XNJ 2 mL·kg^−1^·d^−1^(1d)	CIA, NCA

Zhu 2012	Rat/SD	NR	200	40(20/20)	P	2/168	c.i.v. NS	c.i.v. XNJ 2 mL·kg^−1^·d^−1^(1d)	NDS, CIA, NCA

Huang 2012	Mouse	M	20~22	24(12/12)	P	2/NR	i.p. NS	i.p. XNJ 10 mL·kg^−1^·d^−1^(5d)	CIA, IL-6, IL-1*β*, TNF-*α*

Li and Xie 2011	Rat/SD	M	250~300	12(6/6)	P	2/24	i.v.NS	i.v. XNJ 10 mL·kg^−1^·d^−1^(1d)	NDS, CIA, IL-6, IL-1*β*, BBB

Guo et al. 2010	Rat/SD	M	200~250	16(8/8)	P	2/24	i.v.NS	i.v.10 mL·kg^−1^·d^−1^(1d)	MDA, SOD, NCA

Wu 2007	Rat/W	M	260±10	43(23/20)	T	8/-	i.p.NS	i.p. XNJ 8 mL·kg^−1^·d^−1^(5d)	TNF-*α*, BE

Zhang et al. 2006	Mouse	M&F	50-80	18(9/9)	P	0.16/4	i.v.NS	i.v. XNJ 0.685 mL·kg^−1^·d^−1^ (1d)	SOD

Lu 2006	Rat/SD	M	200~250	16(8/8)	P	2/48	i.p. NS	i.p. XNJ 10 mL·kg^−1^·d^−1^(1d)	SOD, MDA, NCA

Shen et al. 2004	Rat/SD	M	250 ±20	12(6/6)	T	6/-	NR	i.p. 1.25 mL·kg^−1^·d^−1^, 2times(1d)	NDS

Wang et al. 2004	Rabbit	M&F	2200~3000	20(10/10)	P	0.5/2	i.v. NS	i.v. XNJI mL·kg^−1^·d^−1^(1d)	MDA, SOD

Zhou et al. 2002	Mouse	M	28~30	15(7/8)	P	0.08/0.16	NR	i.p. XNJ 5 mL·kg^−1^·d^−1^(1d)	GSH-Px, SOD, LMF

Wan et al. 2001	Rat/SD	M&F	200~250	16(8/8)	T	0.66/-	i.p. NS	i.p. XNJ 3 mL·kg^−1^·d^−1^(1d)	GSH-Px

Cai et al. 2000	Rat/W	M	205±15	20(10/10)	T	3/-	i.p. NS	i.p. XNJ 2 mL·kg^−1^·d^−1^(1d)	NS

Fu et al. 2000	Rat/W	M	200~300	30(15/15)	P	3/3	NR	i.p. XNJ 20 mL·kg^−1^·d^−1^(7d)	BE, CIA, NCA

Chen et al 2000	Rabbit	M&F	2200~3600	20(10/10)	P	0.5/2	i.v. NS	i.v. XNJ 1 mL·kg^−1^(1d)	TNF-*α*, IL-1*β*, IL-6, BUC

Zhang et al 1997	Rabbit	NR	2500~3000	12(6/6)	P	0.5/0.5	NR	i.v. XNJ 40 mL·kg^−1^(1d)	MDA, GSH-Px,

Note. XNJI is Xingnaojing injection; SD is Sprague-Dawley; W is Wistar; M is male; F is female; P is permanent; T is transient; i.p. is intraperitoneal injection; i.v. is intravenous injection; c.i.v. is caudal intravenous injection; NR is not report; Index isch/rep(h) is index ischemia and reperfusion in hours; - means no reperfusion; BE is brain edema; BBB is blood brain barrier; NDS is Neurological Deficit Score; CIA is cerebral infarction area; NCA is Neuronal cell apoptosis; TNF-*α* is brain tumor factor *α*; IL-6 is interleukin-6; IL-1*β* is interleukin-1*β*; SOD is superoxide dismutase; MDA is malondialdehyde; GSH-Px is glutathione peroxidase.

**Table 2:** Quality assessment of included studies.

**Study (year)**	**(1)**	**(2)**	**(3)**	**(4)**	**(5)**	**(6)**	**(7)**	**(8)**	**(9)**	**(10)**	**score**
Yan et al. 2017	√	UN	√	UN	UN	UN	√	√	UN	UN	4
Li et al. 2016	√	UN	√	UN	UN	√	√	√	UN	UN	5
Chen et al. 2015	√	√	√	UN	UN	√	√	√	UN	UN	6
Wu 2015	√	√	√	UN	UN	√	√	√	UN	UN	6
Zhong et al. 2015	√	UN	√	UN	UN	√	√	√	UN	UN	5
Huang et al. 2014	√	UN	√	UN	UN	√	√	√	UN	UN	5
Ma 2014	√	√	√	UN	UN	√	√	UN	UN	UN	5
Zeng 2013	√	√	√	UN	UN	√	√	√	UN	UN	6
Zhu 2012	√	UN	√	UN	UN	√	√	√	UN	UN	5
Huang 2012	√	√	UN	UN	UN	√	√	UN	UN	UN	4
Li and Xie 2011	√	UN	√	UN	UN	UN	√	√	UN	UN	4
Guo et al. 2010	√	UN	√	UN	UN	UN	√	UN	UN	UN	3
Wu 2007	√	UN	√	UN	UN	√	√	√	UN	UN	5
Zhang et al. 2006	√	UN	√	UN	UN	√	√	UN	UN	UN	4
Lu 2006	√	UN	√	UN	UN	√	√	UN	UN	UN	4
Shen et al. 2004	√	√	UN	UN	UN	UN	√	UN	UN	UN	3
Wang et al. 2004	√	UN	√	UN	UN	√	√	√	UN	UN	5
Zhou et al. 2002	√	UN	√	UN	UN	√	√	UN	UN	UN	4
Wan et al. 2001	√	√	UN	UN	UN	√	√	UN	UN	UN	4
Cai et al. 2000	√	UN	√	UN	UN	√	√	√	UN	UN	5
Fu et al. 2000	√	UN	√	UN	UN	UN	√	√	UN	UN	4
Chen et al. 2000	√	UN	UN	UN	UN	UN	√	√	UN	UN	3
Zhang et al. 1997	√	UN	UN	UN	UN	√	√	√	UN	UN	4

Note: (1) publication in a peer-reviewed journal; (2) statement of temperature control;(3) random allocation to groups; (4) allocation concealment; (5)blinded assessment of outcome; (6) use of anesthetic without significant internal protection of blood vessel; (7) appropriate animal model (aged, healthy, diabetic, or hypertensive); (8) sample size calculation; (9) compliance with animal welfare regulations; (10) statement of potential conflict of interests. UN is unclear.

## Abbreviations

XNJI: Xingnaojing injection
TCPM: Traditional Chinese patent medicine
CNKI: Chinese National Knowledge Infrastructure
VMIS: VIP medicine information system
ARRIVE: Animal Research: Reporting of In Vivo Experiments
MD: Mean difference
*CIs*: Confidence intervals
*I*^2^: *I*-square
CONSORT: Consolidated Standards of Reporting Trials
PAF: Platelet activating factor
SD: Sprague-Dawley
W: Wistar
M: Male
F: Female
P: Permanent
T: Transient
i.p.: Intraperitoneal injection
i.v.: Intravenous injection
c.i.v.: Caudal intravenous injection
NR: Not report
Index isch/rep(h): Index ischemia and reperfusion in hours
BE: Brain edema
BBB: Blood brain barrier
NDS: Neurological deficit score
CIA: Cerebral infarction area
NCA: Neuronal cell apoptosis
BUC: Brain ultrastructural changes
TNF-*α*: Brain tumor factor *α*
IL-6: Interleukin-6
IL-1*β*: Interleukin-1*β*
SOD: Superoxide dismutase
MDA: Malondialdehyde
GSH-Px: Glutathione peroxidase.

## References

[1] Kaur I., Kumar A., Jaggi A. S. (2017). Evidence for the role of histaminergic pathways in neuro- protective mechanism of ischemic post-conditioning in mice. *Fundamental & Clinical Pharmacology*.

[2] Zhang Y., Xu H., Sun H. (2014). Electroacupuncture treatment improves neurological function associated with regulation of tight junction proteins in rats with cerebral ischemia reperfusion injury. *Evidence-Based Complementary and Alternative Medicine*.

[3] Liu C., Liu J. X., F Ren F. (2016). Medical doctoral medicine revascularization damage efficiency research advancement. *Chinese Gerontology Journal*.

[4] Wang X., Cui D. Z. (2014). Advances in clinical application of An gong Niu huang Pill in various encephalopathy. *Chinese Journal of Gerontology*.

[5] Krishnamurthi R. V., Feigin V. L., Forouzanfar M. H. (2013). Global and regional burden of first-ever ischaemic and haemorrhagic stroke during 1990–2010: findings from the Global Burden of Disease Study 2010. *The Lancet Global Health*.

[6] Li Y., Liu S. (2017). The effect of dexmedetomidine on oxidative stress response following cerebral ischemia-reperfusion in rats and the expression of intracellular adhesion molecule-1 (ICAM-1) and S100B. *Medical Science Monitor*.

[7] Schilling M., Besselmann M., Leonhard C., Mueller M., Ringelstein E. B., Kiefer R. (2003). Microglial activation precedes and predominates over macrophage infiltration in transient focal cerebral ischemia: a study in green fluorescent protein transgenic bone marrow chimeric mice. *Experimental Neurology*.

[8] Chen X., Wang K. (2016). The fate of medications evaluated for ischemic stroke pharmacotherapy over the period 1995–2015. *Acta Pharmaceutica Sinica B (APSB)*.

[9] Huang X.-P., Ding H., Lu J.-D., Tang Y.-H., Deng B.-X., Deng C.-Q. (2015). Effect of the combination of the main active components of astragalus and panax notoginseng on inflammation and apoptosis of nerve cell after cerebral ischemia-reperfusion. *American Journal of Chinese Medicine*.

[10] Zhao Y. F., Jiang Y. P., Xiao B. G. (2013). New progress of recombinant tissue plasminogen activator in the treatment of acute ischemic stroke. *Chinese Journal of Clinical Neuroscience*.

[11] Candelario-Jalil E. (2009). Injury and repair mechanisms in ischemic stroke: considerations for the development of novel neurotherapeutics. *Current Opinion in Infectious Diseases*.

[12] Pragathi D. G., M Rampure D. D., Sreeram D. G. (2015). Study of Lipid Profile in Ischemic Cerebrovascular Disease. *Journal of Medical Science And clinical Research*.

[13] Huang C. F. (2012). Effect of Xingnaojing injection on serum IL-6 and TNF-a in mice with cerebral ischemia and reperfusion injury. *China Practical Medicine*.

[14] Zhang Y., Duan S. Q., Xie X. D. (2010). The application of XNJ in acute encephalopathy. *Journal of Hei Long Jiang Medicine*.

[15] Gao X. F., Wu Y. S. (2008). Protective effect of Xingnaojing injection on inflammatory injury induced by acute cerebral ischemia in rats. *Practical Medicine Journal*.

[16] Wu Y. S., Zhang W. G., Zhang G. L. (2007). Effect of Xingnaojing injection on inflammatory reaction and brain edema and preparations in rats with acute brain ischemia. *Journal of Shandong University*.

[17] Chen C., Cheng F., Liao S., Chen W., Lin N., Kuo J. (2000). Effects of naloxone on lactate, pyruvate metabolism and antioxidant enzyme activity in rat cerebral ischemia/reperfusion. *Neuroscience Letters*.

[18] Li Y. F. (2007). Effect of Xingnaojing injection on cognitive function in rats with chronic cerebral ischemia. *Practical Medicine Journal*.

[19] Cai Y. M., Xue R. L., Li W. (2004). Effect of global cerebral ischemia-reperfusion on oxygen free radical and endothelin in rats. *Chinese Journal of Rehabilitation Medicine*.

[20] Zhong Z. Y., Li B., Liu M. (2015). Effect of Xingnaojing Injection on expression of ZO-1 in Blood Brain Barrier of rats with cerebral ischemia and reperfusion. *Chinese Journal of Pathophysiology*.

[21] Zhu B. (2012). *Effect of Xingnaojing on GABA-A Receptor after Focal Ischemia Reperfusion Injury [Medicine master's degree thesis]*.

[22] Lu X. W. (2006). *The Protective Effect of Xingnaojing Injection on Cerebral Ischemia Reperfusion Injury in Rats [Medicine master's degree thesis]*.

[23] Zhang H. B. (2006). Experimental study of Xingnaojing against cerebral ischemia-reperfusion Injury in Rats. *Zhejiang Journal of Integrated Traditional Chinese and Western Medicine*.

[24] Fu Q., Cui H. L., Sun Z. J. (2000). The study of prevent effect of Xingnaojing injection in neuronal apoptosis induced by ischemia reperfusion. *Chinese Journal of Integrative Medicine on Cardio/Cerebrovascular Disease*.

[25] Ma X., Yang Y. X., Chen N. (2017). Meta-Analysis for Clinical Evaluation of Xingnaojing Injection for the Treatment of Cerebral Infarction. *Frontiers in Pharmacology*.

[26] Macleod M. R., O'Collins T., Howells D. W., Donnan G. A. (2004). Pooling of animal experimental data reveals influence of study design and publication bias. *Stroke*.

[27] Yan R. Y., Wang S. J., Wang Y. (2017). Effect of xingnaojing injection combined with butylphthalide(NBP) on the expression of apoptosis after cerebral ischemia reperfusion. *Stroke and Nervous Diseases*.

[28] Li C. L., Huang C. F., Zhang F. (2016). Effect of Xingnaojing injection combined with brain activating and orifice-opening acupuncture on inflammatory cytokines in serum and brain tissue of rats with global cerebral ischemia reperfusion injury. *Chinese Journal Clinical Pharmacology*.

[29] Chen J., Li B., Liu M. (2015). The effects of Xingnaojing injection on Caveolin-1 in cortex of brain after global ischemia-reperfusion. *Chinese Journal of Emergency Medicine*.

[30] Wu P. (2015). *Study on Effect of Xingnaojing on Rat Cerebral Ischemia Reperfusion Inflammatory Injury*.

[31] Huang G. H., Yu J. F., Meng H. H. (2014). Effect of Xingnaojing on autophagy of neurons in rats with cerebral ischemia and reperfusion. *Chinese Journal of Traditional Chinese Medicine*.

[32] Ma B., Liu L., Zhang Y. (2014). Xingnaojing injection attenuates neurologic deficits against focal ischemic injury in stroke-prone renovascular hypertensive rat. *Chinese Journal of Integrative Medicine on Cardio/Cerebrovascular Disease*.

[33] Zeng F. J. (2013). *Xingnaoiing on cerebral ischemia reperfusion iniury gene Bcl-2 and Beclin 1 autophagy gene expression [Medicine master's degree thesis]*.

[34] Li F. J., Xie S. L. (2011). Protective effect of Xingnaojing injection on cerebral ischemia-reperfusion injury in rats. *Chinese Traditional Medicine*.

[35] Guo F., Lu X.-W., Xu Q.-P. (2010). Protective effect of Xingnaojing and Xuesaitong injections on cerebral ischemic reperfusion injury in rats. *National Medical Journal of China*.

[36] Wang W. T., Chen S. Q., Wang W. (2004). Effect of Xingnaojing injection on oxygen free radical in experimental cerebral ischemia and reperfusion Injury. *Chinese Journal of Traditional Chinese Medicine*.

[37] Shen S. Y., Gan Z. H., Fu X. D. (2004). Effect of Xingnaojing on neuro behavior of rats with focal cerebral ischemia. *Journal of Hebei Traditional Chinese Medicine*.

[38] Zhou H. Y., Hu G. X., Chen X. Y. (2002). The protective effect of XNJI on memory impaired by cerebral ischemia reperfusion. *Journal of Wenzhou Medical College*.

[39] Wan W. C., Luo H. Y., Chen J. W. (2001). An experiment study of eliminating toxic heat and inducing resuscitation in the treatment of the incomplete cerebral ischemia. *Journal of Shenzhen Combine Traditional Chinese and Western Medicine*.

[40] Cai D., Ruan J., Yu J. (2000). Clinical and experimental study on Xingnaojing injection in treating acute ischemic cerebral apoplexy. *Journal of Emergency in Traditional Chinese Medicine*.

[41] Zhang J., Zhang G. Q., Peng X. Y. (1997). Experimental study on cerebral ischemia reperfusion injury. *Journal of Emergency Medicine*.

[42] Zhang Q. D. (2012). *The Preparation Process and Quality Control of Refined Xingnaojing Injection [Medicine master's degree thesis]*.

[43] Wang J., Yang J.-Q., Yu L.-J., Yang B., Zhao L., Jiang Q.-S. (2014). COX2-PGI2/TXA2 signal pathway involved in protective mechanism of PDTC pretreatment against global cerebral ischemia reperfusion rat hippocampus injury. *Chinese Pharmacological Bulletin*.

[44] Deng L. L., Tian L., Wang H. C. (2010). Research progress on clinical application of An gong Niu huang wan and its derivative prescription. *Chinese Journal of Experimental Traditional Medical Formulae*.

[45] Jiang M., He J., Wang H. (2012). Effectiveness of edaravone combined with Xingnaojing injection for adult acute cerebral infarction: a systematic review. *Chinese Journal of Evidence Based Medicine*.

[46] Chen S. Q., Wang W. T., Wang M. S. (2000). Experimental research of effect of Xingnaojing on changes TNF-, IL-1*β*, IL-6 in plasma and brain tissue and ultrastructure of brain tissue in rabbit with cerebral ischemia- reperfusion. *Chinese Journal of Critical Care Medicine*.

[47] Hakimizadeh E., Shamsizadeh A., Roohbakhsh A. (2017). Inhibition of TRPV1 confers neuroprotection, reduces TNF-a and increases Il-10 in a rat stroke model. *Fundamental & Clinical Pharmacology*.

[48] Gasche Y., Copin J., Sugawara T., Fujimura M., Chan P. H. (2001). Matrix metalloproteinase inhibition prevents oxidative stress-associated blood-brain barrier disruption after transient focal cerebral ischemia. *Journal of Cerebral Blood Flow & Metabolism*.

[49] Feng X., Yang S., Liu J. (2013). Electroacupuncture ameliorates cognitive impairment through inhibition of NF-*κ*B-mediated neuronal cell apoptosis in cerebral ischemia-reperfusion injured rats. *Molecular Medicine Reports*.

[50] Stroke Therapy Academic Industry Roundtable II (STAIR-II) (2001). Recommendations for clinical trial evaluation of acute stroke therapies. *Stroke*.

[51] Michael P. K., Bix G. J. (2012). Successfully climbing the "sTAIRs": Surmounting failed translation of experimental ischemic stroke treatments. *Stroke Research and Treatment*.

[52] Fisher M. (2003). Recommendations for advancing development of acute stroke therapies: Stroke Therapy Academic Industry Roundtable 3. *Stroke*.

[53] Schulz K. F., Altman D. G., Moher D. (2010). CONSORT 2010 statement: updated guidelines for reporting parallel group randomized trials. *Annals of Internal Medicine*.

